# Chronic autoimmune cytopenias in pediatric age group: Clinical features and outcome

**DOI:** 10.1016/j.htct.2026.106540

**Published:** 2026-09-25

**Authors:** Heba G.A. Ali, Hanan M. Abd El Lateef, Sally R.Z. Gallab, Iman A. Ragab

**Affiliations:** a Pediatric Hematology-Oncology, Children`s Hospital, Ain Shams University, Cairo, Egypt; b Pediatric Allergy-immunology and Rheumatology, Children`s Hospital, Ain Shams University, Cairo, Egypt

**Keywords:** Chronic immune thrombocytopenia, Autoimmune hemolytic anemia, Evans syndrome, Autoimmune lymphoproliferative syndrome

## Abstract

**Introduction:**

Immune cytopenias are not uncommon among children. The aim of this study was to describe the clinical and laboratory results, features and treatment responses of children with chronic immune cytopenias.

**Methods:**

A retrospective study of children with chronic immune cytopenias was performed. Clinical presentations, laboratory and immunological results, treatment lines, and responses were reviewed.

**Results:**

A total of 180 patients were included in this study with a median age of 60 months. Chronic immune thrombocytopenia was diagnosed in 47.2 % of the patients, autoimmune hemolytic anemia in 32.8 %, Evans syndrome in 10.6 %, autoimmune lymphoproliferative syndrome in 7.2 % and common variable immunodeficiency in 2.2 % of the cases. Children with autoimmune hemolytic anemia and Evans syndrome had a younger age at presentation and higher lymphocyte counts compared to other groups while no difference was found in immunoglobulin or T-cell subsets. Patients with immune thrombocytopenia had a worse end of study response.

**Conclusion:**

Management of chronic immune cytopenia remains challenging. Chronic immune thrombocytopenia had the worse end of study treatment response. The contribution of genetic predisposition in areas with high consanguinity rates remains to be determined.

## Introduction

Autoimmune cytopenias represent an important and treatable cause of peripheral blood cell cytopenias. These conditions are frequently encountered in pediatric hematology and rheumatology practice. Most children present with unilineage primary disease, which is more common than secondary autoimmune cytopenias. Conversely, patients with chronic or multilineage cytopenias more frequently have an underlying predisposing etiology, warranting a comprehensive diagnostic evaluation [1].

Autoimmune cytopenias include immune thrombocytopenia (ITP), autoimmune hemolytic anemia (AIHA), and autoimmune neutropenia. Chronic ITP is defined as thrombocytopenia persisting beyond 12 months from initial diagnosis. Data from the Intercontinental Cooperative ITP Study Group Registry II indicate that nearly 20 % of enrolled children met the definition for chronic ITP at 12 months post-enrollment [2]. Furthermore, refractory chronic ITP develops at an annual incidence rate of 1.15 % and is significantly associated with older age [3]. Childhood AIHA has an estimated incidence of 0.2 per one million individuals under 20 years of age [4]. In pediatric populations, warm AIHA may occur as a primary disorder or secondary to an underlying systemic illness, often requiring more aggressive therapeutic interventions [5]. Multilineage cytopenia in Evans syndrome can occur either concomitantly or sequentially and is frequently accompanied by lymphoproliferation. Over 40 % of pediatric patients meet the diagnostic criteria for autoimmune lymphoproliferative syndrome (ALPS), and up to 45 % harbor underlying pathogenic variants of immune dysregulation genes [6].

Several underlying pathologies may be encountered in chronic immune cytopenias including ALPS and common variable immunodeficiency (CVID). In ALPS, apart from elevations of CD3+TCRα/β+CD4-CD8- double-negative T cells and chronic lymphoproliferation, the most common autoimmune manifestations are autoimmune cytopenias [7]. While in CVID, more than 33 % of patients present with immune cytopenias, especially Evans syndrome [8].

Overall, most children with immune-mediated cytopenia will have a relatively mild to moderate clinical severity, requiring only close observation with varying combinations of corticosteroids and immunoglobulin therapy [1]. A subgroup of these patients develops unusual disease characteristics such as recurrent, chronic, or refractory cytopenia, multiple cytopenias, cytopenia accompanied by adenopathy and organomegaly, with the possibility of underlying immunodeficiency, or features suggestive of systemic autoimmune or autoinflammatory conditions [9].

Consequently, unexplained cytopenia presents significant diagnostic and management difficulties. This study aimed to describe the clinical, laboratory, and immunological features, as well as treatment responses, in children and adolescents with chronic immune cytopenias.

## Methods

Data from children with a provisional diagnosis of persistent immune cytopenias lasting a minimum of three months, affecting either a single lineage or multiple lineages and followed up at the Pediatric Hematology Unit, Ain Shams University Children’s Hospital, were retrospectively collected. Patients with a proven diagnosis of underlying rheumatological disease were excluded. The study was approved by the local ethics committee of the Pediatric Department, Faculty of Medicine, Ain Shams University.

Diagnostic criteria were defined as follows: chronic ITP was defined as thrombocytopenia persisting for at least 12 months from initial diagnosis [10]; AIHA was defined as anemia (hemoglobin [Hb] level ≤ 2 standard deviations below age- and sex-adjusted means) with a positive direct antiglobulin test and laboratory evidence of hemolysis (reticulocytosis, unconjugated hyperbilirubinemia, and elevated lactate dehydrogenase) [11]; and Evans syndrome was defined as the simultaneous or sequential immune-mediated destruction of two or more hematologic cell lineages [12].

CVID was defined according to European Society of Immune Deficiencies (ESID) and the Pan American Group for Immune Deficiency (PAGID) [13], and ALPS according to 2009 National institutes of Health (NIH) International workshop [14].

Data collected from medical files included age at onset, disease duration, sex, presenting complaints, positive family history of a similar condition or other immunological diseases, consanguinity, and past history including medications and surgeries. Clinical examinations included the bleeding phenotype for ITP, presence of pallor and jaundice, liver and spleen size, and lymph node status. Laboratory results included a complete blood count, reticulocyte count, total and direct bilirubin levels, liver transaminases, lactate dehydrogenase, Coombs test, and viral markers (hepatitis B and C, rubella, cytomegalovirus [CMV], Epstein-Barr virus [EBV], and herpes simplex virus). Immunological evaluation comprised total serum immunoglobulin levels (IgA, IgG, IgM, and IgE) and flow cytometric immunophenotyping, including CD3+, CD4+, CD8+, and CD56+ percentages, the CD4/CD8 ratio, and double-negative αβ T cells. Anticardiolipin antibodies, antiphospholipid antibodies, antinuclear antibody (ANA), anti-double stranded DNA antibody (Anti-dsDNA) were recorded in particular cases. Medical records were also reviewed to evaluate the various lines of therapy administered and the subsequent clinical responses.

### Response criteria of immune cytopenias

For ITP, response criteria in pediatrics can be defined as complete remission (CR) if platelet count ≥100 × 10^9^/L with or without treatment [10]. For autoimmune hemolysis, CR is defined as Hb >12 g/dL and normalization of all hemolytic markers, and partial response (PR) is defined as Hb = 10-12 g/dL or at least 2 g/dL increase in Hb without the requirement of transfusion [15].

### Statistical analysis

Data were analyzed on an IBM-PC compatible computer using Statistical Program for Social Science version 20.0 (SPSS Inc., Chicago, Illinois). Quantitative variables were described as means ± standard deviation for parametric data and as medians and inter-quartile ranges for non-parametric data. Qualitative variables were described as counts and percentages. Inferential analyses were performed for quantitative variables using an independent t-test in cases of two independent groups with parametric data and the Mann-Whitney test in cases of two independent groups with non-parametric data. Inferential analyses were performed for qualitative data using the Chi square test for independent variables. Comparative studies between different groups were made using the log-rank test. A p-value <0.05 was considered significant.

## Results

### Demographic and clinical data

This study included 180 children with chronic cytopenias with a median age of 60 months. Ninety-three (51.7 %) were males and 87 (48.3 %) were females. The studied participants were divided into five groups: Group I included 85 (47.2 %) patients with chronic ITP with ages at onset ranging from 6 months to 9 years and a median age of 5 years. This group comprised 36 (42.4 %) males and 49 (57.6 %) females. Group II included 59 (32.8 %) patients with AIHA. Age at onset ranged from 2 months to 16 years, with a mean age of 2.5 years (presentation range: 10 months to 8 years); the cohort comprised 41 (69.5 %) males and 18 (30.5 %) females. Group III comprised 19 (10.6 %) patients with Evans syndrome (7 males and 12 females). The age at symptom onset ranged from 4 months to 12 years, whereas the age at presentation ranged from 2 to 8 years, with a mean presentation age of 3 years. Group IV included 13 (7.2 %) patients with ALPS. Age at onset ranged from 8 months to 15 years, with a mean age of 7 years (presentation range: 2–8 years); the cohort comprised 8 (61.5 %) males and 5 (38.5 %) females. Group V included 4 (2.2 %) patients with CVID-related immune cytopenias. Age at presentation ranged from 6 months to 10 years, with a mean age of 5 years; the cohort comprised one (25.0 %) male and three (75.0 %) females (Figure 1).

**Figure 1:**
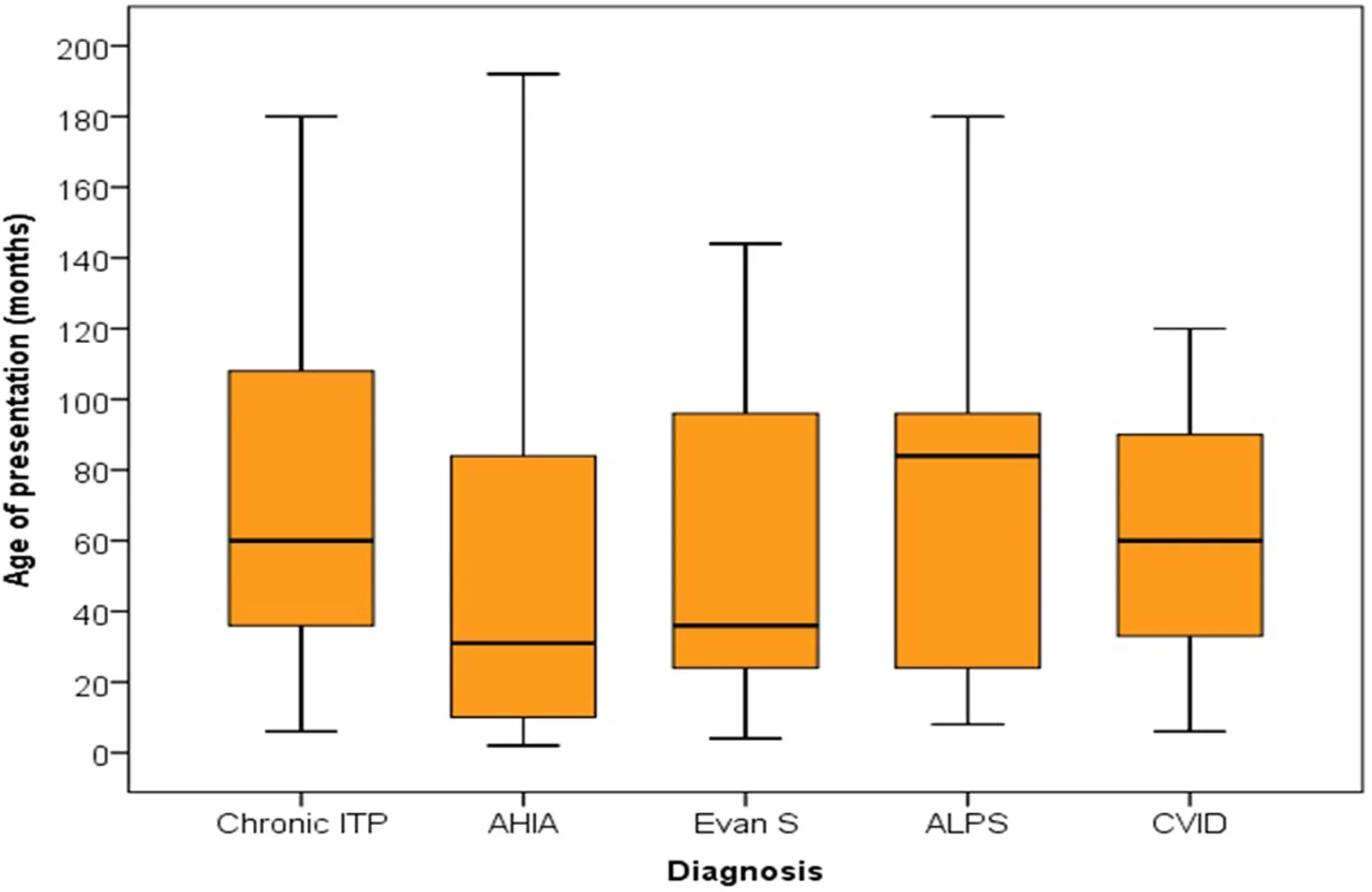
The age at onset of each group of patients ITP: Immune thrombocytopenia; AIHA: Autoimmune hemolytic anemia; Evan S: Evans syndrome; ALPS: Autoimmune lymphoproliferative syndrome; CVID: Common variable immunodeficiency.

Out of the total group, 57 (31.7 %) patients had a history of parental consanguinity, nine (5.0 %) patients had family history of a similar condition, and five (2.7 %) patients had history of other possible immune related conditions in their families (Table 1). Regarding other immune manifestations, one of 85 patients with chronic ITP had vitiligo and one patient with AIHA had autoimmune thyroiditis; details of the other autoimmune diseases were not recorded. Sixty patients (33.3 %) had hepatomegaly, 52 (28.9 %) had splenomegaly and 17 (9.4 %) had lymphadenopathy.

**Table 1:** Demographic data of patients with chronic immune cytopenias.

		Chronic ITP	AIHA	ES	ALPS	CVID
		n = 85	n = 59	n = 19	n = 13	n = 4
Gender - n (%)	Male	36 (42.4)	41 (69.5)	7 (36.8)	8 (61.5)	1 (25.0)
	Female	49 (57.6)	18 (30.5)	12 (63.2)	5 (38.5)	3 (75.0)
Family history of similar condition - n (%)	Positive	6 (7.1)	1 (1.7)	1 (5.3)	1 (7.7)	0 (0.0)
Family history of other immune condition - n (%)	Positive	2 (2.3)	2 (3.3)	0 (0.0)	0 (0.0)	1 (25.0)
Consanguinity - n (%)	Positive	25 (29.4)	18 (30.5)	4 (21.1)	8 (61.5)	2 (50.0)

ITP: Immune thrombocytopenia; AIHA: Autoimmune hemolytic anemia; ES: Evans syndrome; ALPS: Autoimmune lymphoproliferative syndrome; CVID: Common variable immunodeficiency

### Hematological evaluation at presentation

The mean platelet count at diagnosis of patients with chronic ITP was 15 × 10^9^/L (Range: 1–91 × 10^9^/L). Regarding patients with AIHA, the mean Hb level at presentation was 6.2 g/dL (range: 3.2–11.5g/dL), most cases had elevated reticulocyte counts, with a median corrected reticulocyte count of 5.8 % (absolute count: 290 × 10^9^/L) except for two patients who had low corrected reticulocyte counts of 0.5 % (25 × 10^9^/L) and 0.7 % (35 × 10^9^/L). The median total serum bilirubin was 2.9 mg/dL (range: 0.3–11.2 mg/dL) and liver transaminases was normal except for one patient who had hepatitis A.

The initial blood counts in patients with Evans syndrome showed a mean Hb level at presentation of 7.19 g/dL (range: 2.7–12.9 g/dL), average mean corpuscular volume (MCV) of 83.15 fL (range: 60–106.3 fL), the median platelet count was 29 × 10^9^/L (range: 2–33.3 × 10^9^/L), median corrected reticulocyte count was 6 % of red blood cells (range: 0.5–19 %; median absolute count 300 × 10^9^/L), median total serum bilirubin was 1.6 mg/dL (range: 0.4–8.7 mg/dL) and median direct serum bilirubin was 0.4 mg/dL (range: 0.1–4 mg/dL). Significantly higher white blood cell, neutrophil, and lymphocyte counts were detected in children with AIHA and Evans syndrome compared to the other groups (Table 2). In patients with ALPS, the initial mean Hb level at presentation was 7.96 g/dL (range: 3.9–11.7 g/dL), the mean MCV was 79.8 fL (range: 67.6–121 fL), the median corrected reticulocyte count was 3.45 % (range: 0.2–18 %; median absolute count: 172.5 × 10^9^/L), and the median total serum bilirubin level was 1.4 mg/dL (range: 0.1–4.6 mg/dL).

**Table 2:** Hematological and Immunologic parameters of each group of patients.

		Chronic ITP	AIHA	ES	ALPS	CVID
		No. = 85	No. = 59	No. = 19	No. = 13	No. = 4
**White blood cell count** **(x10^9/L)**	**Median (IQR)**	9.3 (6.4 – 11.9)	12.8 (7.8 – 21.9)	9.8 (5.9 – 19.2)	4.8 (3.2 – 6.2)	7.5 (3.5 – 14.5)
	**Range**	2.1–31	3.2–36.5	5.0–28.7	1.13–26.1	2.5–18.5
**Lymphocytes (x10^9/L)**	**Median (IQR)**	3.6 (2.5–4.74)	5.2 (2.3–8.8)	3.9 (3.4–5.46)	2.1 (1.4–2.45)	2.52 (1.6–7.0)
	**Range**	0.9–9.1	0.7–25.5	2.0–5.6	1.3–2.75	1.6–7.0
**Neutrophils (x10^9/L)**	**Median (IQR)**	3.95 (2.75–5.53)	5.95 (4.0–8.2)	3.0 (1.6–4.8)	2.8 (0.55–3.5)	4.72 (2.5–6.93)
	**Range**	0.0–13.2	2.2–19.9	0.5–18.7	0–22.4	2.5–6.93
**IgG (mg/L)**	**Median (IQR)**	920 (639–1265.5)	797.5 (438–1300)	1377 (1142–1612)	2709.5 (1184–3733)	578.2 (394.7–1058.2)
	**Range**	483–2504	310–5270	1142–1612	463–3952	370–1379.4
**IgM (mg/L)**	**Median (IQR)**	98 (75–157)	93 (49–113)	56.5 (27–86)	99.6 (33.75–233.35)	39.5 (32–64.9)
	**Range**	57–400	28–400	27–86	26–309	25–89.8
**IgA (mg/L)**	**Median (IQR)**	86 (82–127)	65 (47.5–114)	154 (81–227)	138 (132–355)	47.35 (17.7–78.65)
	**Range**	22–316	10.5–220	81–227	34–549	15–83
T-Cells (CD3) * (10^9)/L	**Median (IQR)**	2.24 (1.8–2.68)	3.43 (1.29–7.3)	5.34 (5.34–5.34)	1.36 (1.36–1.36)	2.17 (2.17–2.17)
	**Range**	1.8–2.68	1.29–7.3	5.34–5.34	1.36–1.36	2.17–2.17
**CD3%**	**Median (IQR)**	74.1 (72.3–75.9)	69.25 (57.55–70.95)	60.9 (47.3–74.5)	76.3 (67.7–88.7)	73.6 (45–80.2)
	**Range**	72.3–75.9	53.9–74.9	47.3–74.5	67.7–88.7	45–80.2
CD8 * (10^9)/L	**Mean ± SD**	1.12 ± 0.57	2.15 ± 0	1.31 ± 0	0.48 ± 0	0.54 ± 0
	**Range**	0.71–1.52	2.15–2.15	1.31–1.31	0.48–0.48	0.54–0.54
**CD8%**	**Mean ± SD**	48.8 ± 13.29	29.63 ± 7.65	23.65 ± 1.34	33.77 ± 28.59	23.27 ± 10.51
	**Range**	39.4–58.2	19–37.5	22.7–24.6	1.6–56.3	12–32.8
CD4 * (10^9)/L	**Median (IQR)**	1.08 (1.02–1.13)	4.38 (4.38–4.38)	3.37 (3.37–3.37)	0.63 (0.47–0.78)	1.55 (1.55–1.55)
	**Range**	1.02–1.13	4.38–4.38	3.37–3.37	0.47–0.78	1.55–1.55
**CD4%**	**Mean ± SD**	47.95 ± 12.37	38.48 ± 17.41	47.5 ± 22.06	39.27 ± 19.9	51.17 ± 21.31
	**Range**	39.2–56.7	22–62.5	31.9–63.1	17.8–57.1	29–71.5
**CD4 CD8%**	**Median (IQR)**	0.8 (0.7–1.4)	1.2 (1–1.5)	2 (1.4–2.6)	0.9 (0.8–1.6)	2.4 (1.1–2.9)
	**Range**	0.7–1.4	0.3–4.2	1.4–2.6	0.79–23.3	1.1–2.9
**DNT (%)**	**Median (IQR)**	3.49 (1.42–6.52)	1.38 (0.87–2.57)	4.17 (4.17–4.17)	5.6 (2.81–11.3)	4.09 (1.72–16.19)
	**Range**	1.4–7.51	0.51–8.15	4.17–4.17	2.07–24	1.12–26.5

ITP: Immune thrombocytopenia; AIHA: Autoimmune hemolytic anemia; ES: Evans syndrome; ALPS: Autoimmune lymphoproliferative syndrome; CVID: Common variable immunodeficiency; DNT: double negative cells on α /β + T cells

Absolute values are described, however normality data were age related

In patients with CVID, the mean Hb level at presentation was 5.7 g/dL (range: 3.9–7 g/dL), mean MCV was 79.93 fL (range: 72.2–93.6 fL), the median platelet count was 388 × 10^9^/L (range: 190–592 × 10^9^/L), the median corrected reticulocyte was 7.2 % of red blood cells (range: 4–43.7 %; median absolute count 360 × 10^9^/L), the median total serum bilirubin was 1.7 mg/dL (range: 0.6–3.1 mg/dL) and the median direct serum bilirubin was 0.4 mg/dL (range: 0.1–1.3 mg/dL).

### Immunologic investigations

ANA testing was performed in 70 of the 180 patients (38.9 %), yielding positive results in ten cases (14.3 %). Positivity rates by diagnostic group were as follows: 6/40 (15.0 %) with chronic ITP, 2/15 (13.3 %) with AIHA, 1/9 (11.1 %) with Evans syndrome, 0/4 (0 %) with ALPS, and 1/2 (50.0 %) with CVID-related immune cytopenias. Among the 180 patients, 26 (14.4 %) underwent lymphocyte subset analysis (Table 2). Flow cytometric assessment of double-negative T cells (DNTs: CD3+, TCR α/β+, CD4−, CD8−) was performed in 34 patients (18.9 %). An elevated DNT percentage (>2.5 %) was identified in 18 of these 34 patients (52.9 %), all of whom maintained an absolute total T-cell count >0.5 × 10^9^ cells/L.

### Virology studies

Among the 180 patients, 41 (22.77 %) and 44 (24.4 %) patients were tested for EBV and CMV, respectively. EBV IgM was positive in 3/41 patients (7.3 %), one with AIHA and two with ALPS; while CMV (IgM) was positive in 3/44 cases (6.8 %), one had ITP, one had AIHA and one had ALPS. Hepatitis B surface antigen and hepatitis C virus were negative in 18 patients tested; only two patients were tested for rubella, and both were negative, and three patients were tested for herpes simplex virus and were negative.

### Treatment and outcome

Most patients (n = 147; 81.7 %) received corticosteroids as first-line therapy, achieving an approximate 80 % response rate. Second-line and subsequent therapies comprised intravenous immunoglobulin (IVIG), rituximab, and steroid-sparing agents, including sirolimus, cyclosporine A, and mycophenolate mofetil (MMF). The disease-free survival rate ranged from 0.25-168 months with a median of five months. CR was achieved in 104 patients (57.78 %). End of study treatment outcomes are illustrated in Figure 2.

**Figure 2:**
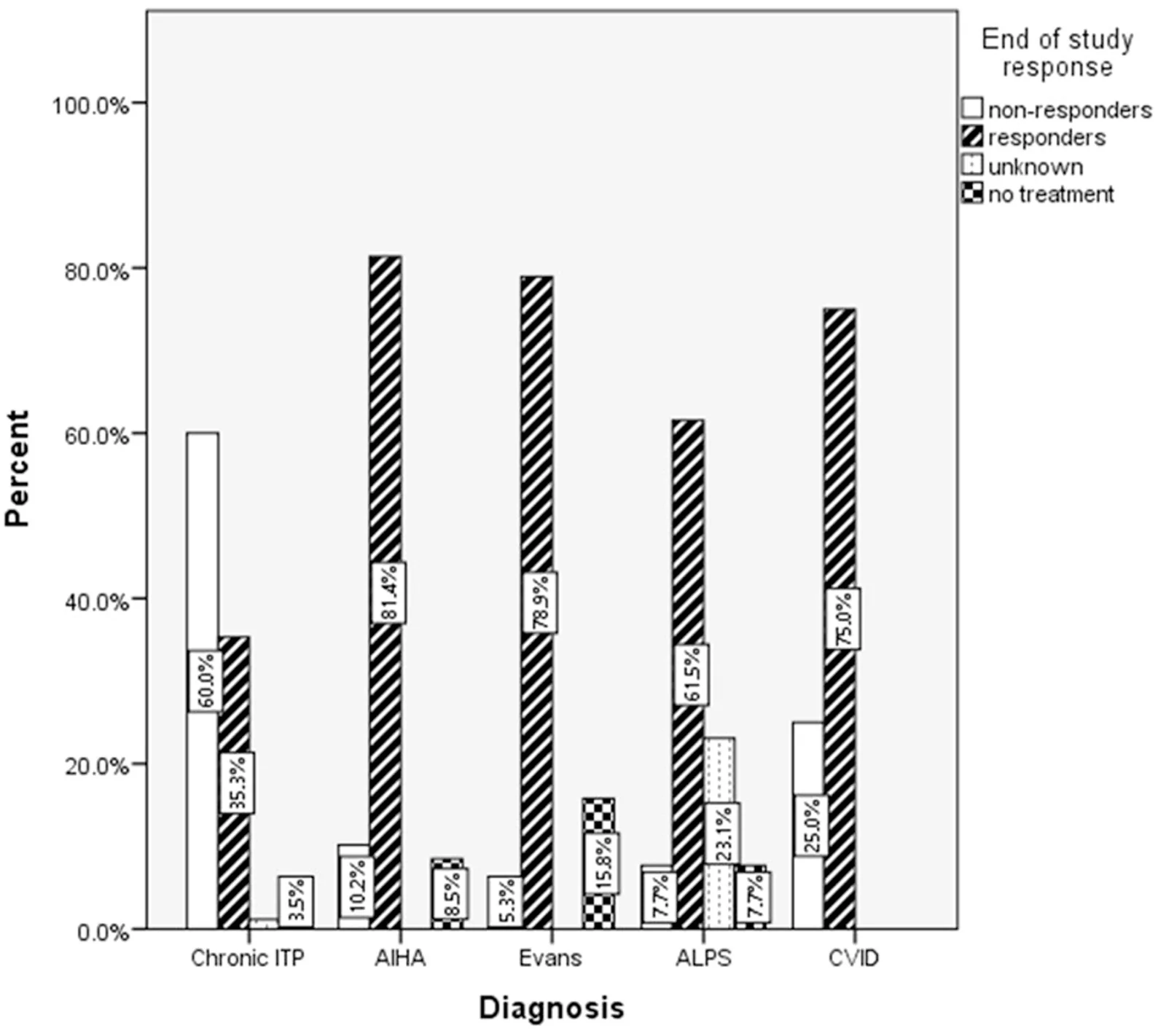
Comparison of different end of study treatment responses in chronic immune cytopenia.

### Immune thrombocytopenia

Patients with ITP received a median of 4 (range: 0–7) different therapies to increase platelet counts. The most common first-line therapy was corticosteroids, IVIG, or a combination of both. Five of 85 patients (5.9 %) did not receive any medical treatment and were managed with watchful observation despite low platelet counts. The median time of CR for patients having chronic ITP was 10.5 months (range: 0.5-168 months) with 30 patients (35.5 %) having good end of study responses to treatment.

### Autoimmune hemolytic anemia

The most common treatment for AIHA was corticosteroids in 51 (86.4 %) patients and IVIG in three (5.1 %) patients. Packed red blood cell transfusions were required in 47 patients (79.7 %); conversely, five patients (8.5 %) required no active intervention and were managed with observation alone. The median time of CR for the patients having AIHA was four months (range: 0.25–108 months) with 48 patients (81.35 %) having good end of study responses to treatment.

### Evans syndrome

Twelve of 19 patients (63.2 %) received steroids, four (21.1 %) received IVIG, and three patients with mild cytopenia were managed with observation alone. Ten patients (52.6 %) had good responses (eight receiving steroid therapy and two receiving IVIG) yet 4/16 (25 %) relapsed and 13 patients (68.4 %) required blood transfusions. Second-line treatments were administered to nine patients (47.4 %), including four with relapsed cytopenia following an initial response, two with primary non-response to first-line therapy, and three managed with extended observation. Second-line regimens comprised steroids in two patients, IVIG in two, CSA in one, and combined therapy in four patients (steroids plus AZA in two, sirolimus in one, and MMF in one). Overall, seven of nine patients (77.8 %) responded to second-line therapy, whereas two patients (10.5 %) required third-line treatments consisting of steroids with AZA and steroids with cyclophosphamide. The median time of response to treatment in this group of patients ranged from 0.25 to 24 months with a median of 3 months; CR was achieved in 15 patients by the end of study (78.9 %).

### Autoimmune lymphoproliferative syndrome

In respect to the first-line treatment, 12 patients (92.3 %) received steroids and one patient was managed without treatment due to mild cytopenia. Seven of the cohort (53.8 %) had good responses and four (30.8 %) relapsed. As a second-line treatment, five (38.5 %) received steroids and MMF, three (23 %) received steroids and sirolimus, one (7.7 %) received MMF alone, one (7.7 %) received steroids and cyclosporin with six patients (46.2 %) having good responses to treatment. As third-line treatment, one patient (7.7 %) received steroids and MMF and one patient received steroids and sirolimus with both showing good responses. The median time of response was 20 months (range 2–60 months) for this group of patients with CR being achieved by eight patients (61.5 %).

### Common variable immunodeficiency

Three of the patients with CVID (75.0 %) received steroids as first-line treatment while one (25.0 %) received Anti-dsDNA; the four patients showed good response to treatment but all of them relapsed so second-line treatment was administered: one patient (25.0 %) received steroids again, one patient (25.0 %) received steroids and MMF, one patient (25.0 %) received steroids and cyclosporin A and the other (25.0 %) received IVIG with only one patient (25.0 %) having a good response. As regards to third-line treatment, two patients (50.0 %) received steroids and sirolimus while one patient (25.0 %) received steroids and MMF; two patients (50.0 %) had a good response. Fourth-line treatment was administered to one patient who received steroids and cyclosporin A but with a bad response. The median time of response was 14.5 months (range: 6–23 months).

There was no difference in sex, age at presentation, family history of similar conditions or of immune conditions or consanguinity between patients with APLS and those with CVID (p >0.05). There was also no difference in viral markers or the laboratory results except for the platelet count, which was lower in ALPS patients (median: 108 × 10^3^/µL; range: 5-470 × 10^3^/µL), compared to CVID (median: 388 × 10^3^/µL; range 190–592 × 10^3^/µL; p = 0.031). Immunoglobulin A levels were significantly lower in patients diagnosed with CVID with a median of 47.35 g/L (range: 17.7–78.65 g/L) compared to those with ALPS with a median of 138 g/L (132–355 g/L; p <0.05) (Table 3).

**Table 3:** Comparison between patients with ALPS and those with CVID as regards to hematological and immunological parameters.

		ALPS	CVID	p-value
		n = 13	n = 4	
Hb (g/dL)	Mean ± SD	7.96 ± 2.75	5.7 ± 1.33	0.139
	Range	3.9–11.7	3.9–7	
HCT (%)	Mean ± SD	30.42 ± 5.33	24.1 ± 4.1	0.199
	Range	23.2–36.4	21.2–27	
MCHC (g/dL)	Mean ± SD	31.73 ± 1.2	28.8 ± 0	0.072
	Range	30–33.6	28.8–28.8	
MCH (pg)	Mean ± SD	26.52 ± 7.57	25.9 ± 4.81	0.916
	Range	22–46	22.5–29.3	
MCV (fL)	Mean ± SD	79.8 ± 16.19	79.93 ± 11.87	0.990
	Range	67.6–121	72.2–93.6	
TLC* (10^9^)/L	Median (IQR)	4.8 (3.2 – 6.2)	7.5 (3.5 – 14.5)	0.497
	Range	11.3–26.1	2.5–18.5	
Lymphocytes * (10^9^)/L	Median (IQR)	2.1 (1.4–24.5)	2.52 (1.6–7.0)	0.253
	Range	1.3–2.75	1.6–7.0	
Neutrophils * (10^9^)/L	Median (IQR)	2.8 (0.55–3.5)	4.71 (2.5–6.93)	0.480
	Range	0–22.4	2.5–6.93	
PLT * (10^9^)/L	Median (IQR)	108 (50 – 192)	388 (211 – 568)	0.031
	Range	5–470	190–592	
T-Cells (CD3) * (10^9^)/L	Median (IQR)	1.36 (1.36–1.36)	2.17 (2.17–2.17)	0.317
	Range	1.36–1.36	2.17–2.17	
CD3%	Median (IQR)	76.3 (67.7–88.7)	73.6 (45–80.2)	0.513
	Range	67.7–88.7	45–80.2	
CD8%	Mean ± SD	33.77 ± 28.59	23.27 ± 10.51	0.583
	Range	1.6–56.3	12–32.8	
CD4 * (10^9^)/L	Median (IQR)	0.63 (0.47–0.78)	1.55 (1.55–1.55)	0.221
	Range	0.47–0.78	1.55–1.55	
CD4%	Mean ± SD	39.27 ± 19.9	51.17 ± 21.31	0.519
	Range	17.8–57.1	29–71.5	
CD4 CD8%	Median (IQR)	0.9 (0.8–1.6)	2.4 (1.1–2.9)	0.297
	Range	0.79–23.3	1.1–2.9	
CD56 (%)	Median (IQR)	0.97 (0.54–1.4)	1.38 (1.01–15)	0.564
	Range	0.54–1.4	1.01–15	
DNT (%)	Median (IQR)	5.6 (2.81–11.3)	4.09 (1.72–16.19)	0.602
	Range	2.07–24	1.12–26.5	

ALPS: Autoimmune lymphoproliferative syndrome; CVID: Common variable immunodeficiency; Hb: Hemoglobin; HCT: Hematocrit; MCHC: Mean corpuscular hemoglobin concentration; MCH: Mean corpuscular hemoglobin; MCV: Mean corpuscular volume; TLC: Total leukocyte count; PLT: Platelet; DNT: double negative cells on α /β + T cells

P-value > 0.05: Non-significant; P-value < 0.05: Significant; P-value < 0.01: Highly significant

≠: Mann Whitney test

## Discussion

In the current study, patients with AIHA and Evans Syndrome were younger at onset compared to those with chronic ITP. There was a predominance of males among patients with AIHA, whereas females predominated in chronic ITP and Evans syndrome. However, no molecular screening for underlying hereditary immunologic disorders was carried out. In a retrospective study screening for primary immunodeficiency in 154 patients with autoimmune cytopenias, there was no difference in age at onset or sex distribution between patients proven to have an underlying immunodeficiency and those without [16]. The underlying immunodeficiencies were DiGeorge syndrome, common variable immunodeficiency, and combined immunodeficiency (either genetically defined or uncharacterized).

A high frequency of consanguineous parents was found in patients with ALPS (61.5 %) and CVID (50 %). In a French national observational study of autoimmune hemolytic anemia, 8 % of studied patients had consanguineous parents. Although no underlying genetic predisposition was formally confirmed, the authors suspected its presence in this group, particularly among patients with a positive family history of autoimmune disease [17].

Out of the total cohort, nine patients (5.0 %) had a family history of a similar condition, and five patients (2.8 %) had a history of other possible immune-related conditions in their family. Out of 85 patients with chronic immune thrombocytopenia, six (7 %) patients had positive family history of ITP. Autoimmune disease overlap should be considered. In the OBS'CEREVANCE study, out of 1609 children with chronic immune thrombocytopenic purpura, autoimmune hemolytic anemia, or Evans syndrome, 15 were diagnosed with inflammatory bowel disease (IBD). An age at autoimmune cytopenia onset exceeding ten years was identified as the sole risk factor for IBD development [18]. The subgroup of immune‐mediated cytopenias is atypical and as patients develop unusual disease characteristics such as recurrent, chronic, or refractory cytopenias; multiple cytopenias (either concurrently or sequentially); cytopenias accompanied by adenopathy and organomegaly; evidence of clinical immunodeficiency; or features suggestive of systemic autoimmune conditions. In the current cohort, 60 patients (33.3 %) had hepatomegaly, 52 (28.9 %) had splenomegaly and 17 (9.4 %) had lymphadenopathy. This group of patients needs to be investigated for underlying inborn errors of immunity.

Regarding autoimmune markers, ANA was positive in 14.3 % of patients tested. The significance of isolated ANA positivity in patients with isolated immune cytopenias remains to be determined. In a French cohort of children with ANA-associated autoimmune cytopenias, ANA was positive in 20 % of children and at a median follow-up of 5.8 years, 22 % of them developed systemic lupus erythematosus, 20 % developed chronic immune thrombocytopenic purpura, 19 % developed autoimmune hemolytic anemia, and 45 % developed Evans syndrome [20]. The authors found that ANA positivity within the first three months after a diagnosis of autoimmune cytopenias, age >10 years at diagnosis, and an ANA titer >1:160 were associated with the subsequent occurrence of systemic lupus erythematosus.

Upon evaluating immunoglobulin levels and lymphocyte subsets in the current cohort, several patients with chronic ITP or AIHA exhibited low immunoglobulin levels. In a study of inborn errors of immunity in patients with autoimmune cytopenias, the prevalence reached 42 %, 64 %, and 62 % in patients with ITP, AIHA, and Evans syndrome, respectively [21]. The underlying diagnoses were common variable immunodeficiency, autoimmune lymphoproliferative syndrome, DiGeorge syndrome, and unclassified inborn errors of immunity. In the same study, significant differences were observed between patients with and without inborn errors of immunity regarding T/B lymphopenia, decreased naïve T cells, reduced percentages of switched memory B cells and plasmablasts, altered immunoglobulin levels, and increased percentages of effector/central memory T cells. With advancements in molecular diagnostic testing, many underlying hereditary immunodeficiencies and immune dysregulation disorders have been elucidated [19]; However, there is difficulty in distinguishing between primary and secondary autoimmune cytopenias because many genetic mutations and polymorphisms predisposing patients to autoimmune cytopenias are still being identified [1].

In the current study, no significant differences were found regarding demographic data, clinical manifestations, viral markers or laboratory tests except for the platelet count between patients with ALPS and CVID. Most results demonstrated an overlap between the clinical features of ALPS and CVID, highlighting the need to consider both conditions in the differential diagnosis of these patients [22].

Different therapeutic modalities were applied using a step wise strategy according to the clinical presentation, institutional protocol of treatment, the underlying etiology, severity and the response criteria. In this setting, glucocorticoids and IVIG were the primary initial treatments across all groups, administered either complementarily or sequentially. Some patients received up to a third-line treatment and combination therapies in cases of partial response, no response and relapse during the study. Relapsed patients represent 38.8 % in chronic ITP, 5.3 % in Evans syndrome, 6.8 % in AIHA, 30.8 % in ALPS, in addition to all the four patients in the CVID group. At the end of the study period, responders constituted 35.5 % of patients with chronic ITP, 81.4 % of those with AIHA, 78.9 % with Evans syndrome, 61.5 % with ALPS, and 75.0 % with CVID, showing significant differences in response rates and time to achieve response. The most employed second-line options in this study were thrombopoietin receptor agonists (TPO-RA) and rituximab in patients with chronic ITP. TPO-RA and rituximab, have response rates of more than 60 % and ∼60 %, respectively [23]. Third-line management including mycophenolate mofetil, sirolimus, cyclophosphamide and cyclosporine A were administered in the ALPS and CVID groups either as a single agent or in combination with steroids. In previous American Society of Hematology (ASH) guidelines for ITP, initial response rates for the third-line treatment include purine analogues (azathioprine, mercaptopurine), mycophenolate mofetil, sirolimus, cyclophosphamide, cyclosporine A, dapsone, danazol and vincristine range from around 30–60 % [24]. Having the highest frequency of end-of-study non-responders, refractory chronic immune thrombocytopenia presents one of the most challenging scenarios within ITP management. The term ‘refractory ITP’ actually covers two distinct clinical presentations as outlined in the recent Intercontinental Cooperative ITP Study Group (ICIS) proposal: one for newly diagnosed cases that do not respond to initial treatment and the other for chronic cases that remain treatment-resistant [25].

Identifying underlying immune dysregulation disorders to guide targeted treatment is crucial, yet access to molecular testing remains limited in resource-constrained settings. Patients who were postulated to have primary ITP without a typical response to therapies or an unexpected disease course may carry novel genetic alterations resulting in loss of immune tolerance. Consequently, treatment regimens can be refined based on genetic testing results, rather than relying on sequential empirical trials of broad-spectrum immunosuppressive agents. For instance, abatacept has been used in cases of CTLA-4 haploinsufficiency and LRBA deficiency [26]; sirolimus, an mTOR inhibitor, has shown efficacy in treating PI3Kδ syndrome [27]; while ruxolitinib (a JAK inhibitor), tocilizumab (an IL-6 inhibitor), and ABT-737 (which targets the anti-apoptotic protein Bcl2) are being applied in settings of STAT1 and STAT3 gain-of-function mutations [28]. Additionally, a number of new agents currently used in adult ITP management, such as neonatal Fc receptor antagonists (like efgartigimod and rozanolixizumab), fostamatinib (a spleen tyrosine kinase inhibitor), and avatrombopag (an oral TPO-RA undergoing pediatric clinical trials), may eventually prove beneficial for children as well.

For pediatric Evans syndrome, most patients required second-line treatments. The rapid initial increase in second-line treatments is partly due to the high rate of early relapse and the current practice of administering steroid-sparing agents to treat them [29]. The use of second-line therapies in a long-term follow-up study increased dramatically with age particularly after the first decade with the most used being rituximab and mycophenolate in 36 % and 19 % of cases, respectively [30]. Numerous studies report that Evans syndrome may represent an early stage in the development of ALPS, an underlying primary immunodeficiency, an autoimmune disorder, or an underlying malignancy, and is frequently associated with single or combined immunopathological manifestations. The treatment landscape for immune cytopenias has evolved significantly, integrating both established therapies and novel targeted approaches. Thus, it is a necessity to develop targeted therapies aimed at the different pathophysiological mechanisms of the disease and specific tests that can indicate the optimal treatment options in a tailored personalized manner.

In conclusion, chronic immune cytopenias are a divergent group with variable initial and late response to treatment. Chronic ITP had the worst long-term response to treatment. Underlying immune dysregulation like ALPS and CVID could be detected in a small group of patients, however underlying molecular diagnosis in regions with high consanguineous rates are becoming an important adjuvant diagnostic tool to proper tailor treatment lines.

## Statements and declarations

### Ethics approval

The study was approved from the ethics committee, Faculty of Medicine, Ain Shams University.

### Funding

This research did not receive any specific grant from funding agencies

## Conflicts of interest

Authors declare no conflicts of interests.

## Data Availability

The data that support the findings of this study are available from the corresponding author upon reasonable request.
